# Engaging with Aging: A Qualitative Study of Age-Related Changes and Adaptations

**DOI:** 10.1093/geroni/igab046.3486

**Published:** 2021-12-17

**Authors:** Shaoqing Ge, Kuan-Ching Wu, Hillary Frey, Maryam Saudagaran, Derick Welsh, Janet Primomo, Basia Belza

**Affiliations:** 1 University of Washington School of Nursing, Seattle, Washington, United States; 2 University of Washington School of Nursing, University of Washington School of Nursing, Washington, United States; 3 University of Washington School of Nursing, University of Washington, Washington, United States; 4 University of Washington Tacoma School of Nursing & Healthcare Leadership, University of Washington Tacoma, Washington, United States; 5 University of Washington, Seattle, Washington, United States

## Abstract

Engaging with Aging is an emerging framework proposed by Carnevali which provides a new lens to understand an active, conscious daily living process of coping with age-related changes (ARCs) taken on by older adults. Study aims were to 1) describe the ARCs experienced by community-dwelling older adults; 2) identify the strategies and resources used by older adults to accommodate the daily living challenges caused by the associated ARCs; and 3) evaluate the framework of EWA based on findings from aims 1 and 2. We conducted semi-structured interviews with 29 participants aged 64 to 98 online due to COVID-19 restrictions. We used a virtual card sort to assist data gathering. Fifteen ARCs (e.g., changes in hearing, changes in stability, changes in sleep, etc.) were mentioned by participants and their corresponding adaptations were discussed. We found that older adults linked their adaptations to their ARCs based on their changing capacities and needs. Commonly used adaptations included conserving energy, utilizing tools or technology, and being more conscious before and while taking actions. The challenges caused by COVID-19 in implementing the adaptations were also discussed (e.g., increased difficulty in understanding others due to mask-wearing). Our study substantiates the EWA framework by showing the commonality among older adults in linking ARCs with adaptations. Implications for clinicians and researchers include using EWA to help older adults identify personalized solutions that fit their capacities. Our study is late-breaking as we recently finished data analysis and the information included was not yet available by the previous submission deadline.

